# Factors influencing psychological concerns about COVID-19 in South Korea: direct and indirect damage during the early stages of pandemic

**DOI:** 10.1186/s12889-023-17476-9

**Published:** 2024-01-02

**Authors:** Min-sung Kim, Sung-il Cho

**Affiliations:** 1 Department of Public Health Science, Graduate School of Public Health, Seoul National University, Seoul, Republic of Korea; 2 Institute of Health and Environment, Seoul National University, Seoul, Republic of Korea

**Keywords:** COVID-19, Pandemic, Psychological concern, Mental health, Korea

## Abstract

**Background:**

COVID-19 pandemic has led to psychological concerns, the distribution of which across populations may differ depending on whether pandemic-related damage is direct or indirect. This study aims to investigate concerns associated with direct and indirect damage according to population characteristics, and identify relatively vulnerable groups that are particularly affected by concerns.

**Method:**

This cross-sectional study used data from the 2020 Korea Community Health Survey, which collected data based on a complex sampling design. A total of 208,106 responses from individuals aged ≥ 19 were collected via in-person interviews. The items related to COVID-19 concerns were measured by Likert scales ranging from 1 to 5 and categorized into two types: direct concerns, which pertained to infection or death, and indirect concerns, which pertained to criticism, vulnerability, and economic damage, through factor analysis. We compared the means and effect size of direct concerns, indirect concerns, and overall concerns using weighted mean, ANOVA, and multiple regression analysis.

**Results:**

Exploratory and confirmatory factor analyses supported a two-factor structure for psychological concerns about COVID-19 (CFI = 0.99, TLI = 0.97, SRMR = 0.02, RMSEA = 0.06), which were divided into direct and indirect concerns. Mean scores were 3.62 for direct concerns and 4.07 for indirect concerns. Direct concerns were higher in females (B = .26); the elderly (B = .15); those diagnosed with hypertension or diabetes (B = .04; B = .06); those with few assistants during quarantine (B = .15); and those whose neighbors responded inappropriately to COVID-19 (B = .07). Indirect concerns were lower among the elderly (B = -.04), and higher among young; married (B = .25); pink- or blue-collar workers (B = .08; B = .06); and those who felt that the city responded inappropriately to COVID-19 (B = .02).

**Conclusion:**

The prevalence of concerns regarding direct and indirect damage caused by the COVID-19 pandemic differed according to population characteristics. Some factors had a marked influence on direct and indirect concerns. Our findings could inform psychological interventions and policies for future pandemics. Customized interventions are needed to prevent negative psychological concerns and improve mental health.

## Background

### Mental health impact of COVID-19

COVID-19, caused by severe acute respiratory syndrome coronavirus-2 (SARS-CoV-2), was first detected in late December 2019 in Wuhan Province, China [1]. The World Health Organization (WHO) named this highly infectious disease as COVID-19 on February 11, 2020, and later declared a pandemic.

In response to the spread of COVID-19, almost all countries implemented various measures to prevent or reduce the rapid spread of the virus, such as social distancing, lockdown, and isolation of infected or at-risk persons. Although these policies can decrease the rate of infection, reduced contact with family, friends, and other social support systems leads to severe mental health issues [2]. Most of the problems that occurred in the wake of COVID-19, such as social stigma [3], economic damage due to declining income [4], and anxiety caused by misinformation on social media [5], worsen mental health.

### Characteristics associated with mental health impact of COVID-19

Previous studies have been reported several characteristics associated with the mental health impact of COVID-19. Lower socioeconomic status, income, and education levels have been found to significantly increase the level of concern about COVID-19 [6]. The presence of assistants during COVID-19-related quarantine has been reported to moderate the relationship between subjective health and psychological concerns about COVID-19 [7]. Furthermore, changes in daily life caused by pandemic, such as restricted outdoor activities [8], and difficulties accessing healthcare services [9, 10], have contributed to increase negative emotions. Individuals with poor subjective health level [11], pre-existing chronic diseases like diabetes, hypertension, and cardiovascular disease [12], and perception of inadequate governmental response have shown heightened levels of fear about COVID-19 [13].

Gender, age, and marital status have been found to influence perception of health risks related to novel viruses, with women, older individuals, and married individuals showing particularly high levels [14], indicating a higher vulnerability to direct damage from COVID-19. Other studies have suggested that mass media including social media platforms, play key role in shaping health risk perception [15, 16].

As the spread of COVID-19 extended from densely populated urban areas to surrounding regions, larger cities with higher population densities became hotspots for the virus [17]. Considering that large-scale outbreaks occurred in the Daegu and Gyeongbuk, South Korea, which led to an increase in stigma and anxiety in these regions [18–21], and that the widespread expression of regional hatred through social media has had consequences resulting in stigmatization [22], the region where an individual resides could significantly impact their mental health. As such, the mental health impact of COVID-19 has been severe and widespread across population.

### Psychological concern as indicator of the mental health

To assess mental health impact of COVID-19, psychological symptoms such as concern, worry, and fear have served as important indicators. Among them, especially concern has been well documented to reflect mental health. In a Canadian cohort, COVID-19-related concerns were risk factors for anxiety disorder and predicted the severity thereof [23]. Greater concern over COVID-19 was strongly associated with mental disorders such as adjustment disorder, anxiety, and posttraumatic stress disorder (PTSD) [24]. Also, concern was the earliest indicator of psychological disorders associated with COVID-19, including generalized anxiety, stress, and PTSD-like symptoms [25].

### Concerns caused by direct and indirect damage of pandemic

Accordingly, many studies have been conducted to explore concerns, and some of them attempted to subdivide concerns into each cause, such as reduced social contact, childcare, and job security [26]. In addition, several studies have reported distinct categories of concerns related to COVID-19 among specific population groups. For instance, in a study investigating adolescents’ concerns about COVID-19 [27], a principal component factor analysis revealed two distinct factors. One factor was associated with concerns about social activities, while the other factor pertained to concerns about getting sick. Similarly, in a study examining COVID-19 concerns among healthcare workers [28], an analysis of the items used to assess their concerns revealed that these concerns could be classified into three primary factors: the risk of infection, work-related challenges, and societal changes. However, the distribution of direct and indirect concerns about pandemic across populations is unclear. Understanding these types of concerns is important as they provide valuable insights into the degree and likelihood of both direct damage caused by the disease itself and indirect damage associated with social aspects resulting from COVID-19.

The need for prevention strategies and interventions targeting mental health is increasing, but policies may not be effectively implemented due to limited financial and human resources. Therefore, it is necessary to investigate which types of damage have exerted a particularly significant impact, depending on the characteristics of population groups. This exploration could serve as the foundation for establishing intervention priorities that take into account the needs of each group.

### Aims of the study

We assumed that the factors mentioned above are characteristics of vulnerable groups who may be more susceptible to direct or indirect damage from COVID-19, and therefore, we purposed to analyze the impact of direct and indirect concerns on these factors. By understanding the psychological consequences of direct and indirect damage caused by COVID-19, it will enable to establish response strategies and systems to modulate controllable risk factors. This study will thus help to minimize the psychological damage caused by COVID-19 and future infectious diseases.

This study aims to evaluate differences in the distribution of concerns about direct and indirect damage across populations, analyze factors influencing concerns, and identify relatively vulnerable groups. For this purpose, we performed several validation processes to confirm the appropriateness of dividing psychological concerns into direct and indirect categories.

## Methods

### Study population and procedures

This cross-sectional study used data from the Korea Community Health Survey (KCHS) conducted by Korea Disease Control and Prevention Agency from August 16 to October 31, 2020. This survey collected data through in-person interviews with adults aged ≥ 19 years. The KCHS used resident population data from the Ministry of Public Administration and Security and housing data from the Ministry of Land, Infrastructure, and Transport, which are representative of the Korean population.

Data from 2020, when COVID-19 was not under control, were analyzed based on evidence that concern is an early indicator of the psychological effects of the pandemic [25]. Of the total of 229,269 responses, 208,106 without missing values were used in the analysis.

## Measures

### Dependent variable

Psychological concerns about COVID-19 were measured by five items. ‘Concerns about infection’ was measured by the question “I’m concerned that I’ll get infected with COVID-19”, ‘Concerns about death’ by “I’m concerned that I’ll die if I get infected with COVID-19”, ‘Concerns about criticism’ by “I’m concerned that if I get infected with COVID-19, I’ll be criticized by others around me”, ‘Concerns about the vulnerable’ by “I’m concerned that vulnerable people in my family (the elderly, infants, and patients) may get infected with COVID-19”, and ‘Concerns about economic damage’ by “I’m concerned that the COVID-19 pandemic will cause economic damage (including loss of a job or difficulty in getting a job)”.

Each item was measured on a 5-point Likert scale (1 point for ‘strongly disagree’, 2 points for ‘somewhat disagree’, 3 points for ‘not sure’, 4 points for ‘somewhat agree’, and 5 points for ‘strongly agree’).

We classified the items into direct and indirect concern categories. Concerns about infection and death are direct concerns because they arise from the direct damage caused by COVID-19. Concerns about criticism, the vulnerable, and economic damage are indirect concerns because they are social aspects relate to consequences that emerged in the aftermath of the pandemic. The direct, indirect, and overall concerns scores were calculated by summing the scores of individual items in each category and dividing by the total number of items, resulting in a range of 1 to 5. Higher scores indicate higher levels of concern. Validation processes of measurement method are presented in the Result.

### Independent variables

Independent variables were divided into three main categories: sociodemographic variables, health-related variables, and COVID-19-related variables.

The sociodemographic variables were sex, age, occupation, annual household income, education, marital status, and region. Health-related variables were subjective health level, subjective stress level, hypertension diagnosis, diabetes diagnosis, and annual unmet healthcare needs. COVID-19-related variables included daily life changes associated with COVID-19, the number of assistants during quarantine due to COVID-19, and the appropriateness of the COVID-19 response of the government, city, mass media, and neighbors. The selection criteria for each variable included in each category were based on the scientific findings of previous studies described previously.

The detailed categories for each sociodemographic variable are as follows. The original continuous variables for age and annual household income were categorized in this study. Age was divided into three groups (19–39, 40–59, and ≥ 60) based on tertiles, while income was categorized using quantiles (≤ 1800, ≤ 3600, ≤ 6000, and > 6000). Occupation categories included white collar (managers, professionals, and clerical workers), pink collar (service and sales workers), and blue collar (agricultural, forestry, and fishery workers, technicians, machine operators and assemblers, elementary workers, and military personnel). Education levels were grouped as ‘middle or low’ (elementary school, village (house) school, or middle school), ‘high school’, and ‘college or over’ (2-, 3-, or 4-year colleges or graduate schools). Regions were classified into the metropolitan area (Seoul, Gyeonggi Province, and Incheon) and other administrative regions (Jeolla Province, Gyeonsang Province, Cyungcheoung Province, Gangwon Province, and Jeju Island), based on South Korea’s administrative division criteria.

In health-related variables, subjective health and subjective stress were measured on Likert scales (5-point and 4-point, respectively) and were reclassified into categories of good and poor, high and low. Annual unmet healthcare needs were defined as the desire for treatment over the past year but being unable to receive it. This was categorized as ‘yes’, ‘no,’ and ‘not applicable (never needed medical care).

In COVID-19 related variables, changes in daily life related to COVID-19 were originally measured on a scale of 0 to 100 with 10-point intervals (0 represents complete suspension of daily life and 100 represents no change at all), and were reclassified into severe (0–40 points), moderate (50–60 points), and mild or none (70–100 points) categories based on the distribution. The number of assistants for urgent help during COVID-19 quarantine, excluding family members living together, was categorized as 0, 1–2, 3–5, and 6 or more people. Variables indicating the appropriateness of COVID-19 response were classified as good, moderate, and poor.

### Statistical analysis

The validation processes conducted to confirm the appropriateness of measuring concerns about COVID-19 as direct concerns and indirect concerns were as follows: exploratory factor analysis, confirmatory factor analysis, and the item-total correlation analysis. A detailed description of each analysis was provided in the Result.

The KCHS is based on a complex sampling design, which requires consideration of weights, stratification variables, and cluster variables. To prevent overestimation of significance, we used normalized weights by dividing each individual raw weight by its mean [29, 30], and the final mean value of all individual weights was adjusted to 1.

In the descriptive analysis, the number of respondents was presented as an unweighted value, but the proportion was presented considering weights. The weighted mean of direct and indirect concerns was calculated for each variable. ANOVA was performed using weights to assess the significance of differences in the mean concern values for each variable. Multiple regression analysis was conducted to verify whether there was a difference in effect size between direct and indirect concerns for each variable. To evaluate the importance of the independent variables in the multiple regression model, all possible sub-models were created and the average increase in R^2^ value when one independent variable was added was calculated, as described previously [31]. All statistical analyses were performed using R software (ver. 4.2.2; R Development Core Team, Vienna, Austria) with svydesign, svytable, svymean, svyvar, and svyglm packages.

## Results

### Validation of measurement method

#### Exploratory factor analysis

For the five items, the overall Kaiser–Meyer–Olkin value was 0.79, and Bartlett’s sphericity test was significant (χ^2^ = 257957.5, df = 10, *p* < 0.001). Skewness and kurtosis of the five items were confirmed to verify the normal distribution assumption. When the absolute values of skewness for the research variables are less than 3.0, and the absolute values of kurtosis are less than 10.0, it is considered to meet the assumption of a normal distribution [32, 33]. In this study, skewness and kurtosis for the five items fall in the range of -1.5 to + 1.5. Therefore, to investigate and analyze the structure of the concerns model, EFA was performed.

The five items were subjected to maximum-likelihood and varimax rotation. The number of factors was determined based on the Kaiser-Guttman criterion (eigenvalue > 1 rule), Scree test, parallel analysis (PA), and comparison data (CD) analysis. The Kaiser-Guttman criterion supported a one-factor solution (eigenvalue = 2.63), and this was confirmed by the Scree test. However, PA using 1,000 random datasets and a 95% cutoff suggested a two-factor solution, as did the CD analysis. Combining PA and CD with a descriptive measure is recommended to confirm the number of factors [34], and considering the theoretical justification for factor interpretability based on the distinction between direct and indirect damage related to the pandemic, as outlined in the introduction, we ultimately used a two-factor solution.

Statistically meaningful loadings were assessed by poor (0.32), fair (0.45), good (0.55), very good (0.63), and excellent (0.71) [35, 36]. The range of factor loading values was applied from fair to excellent level, according to which we classified the direct and indirect concerns (Table 1).

**Table 1:** Results of exploratory factor analysis of concerns about COVID-19

	Factor loading	
Item	Direct concerns	Indirect concerns
Concerns about infection	**0.74**	0.33
Concerns about death	**0.67**	0.29
Concerns about criticism	0.44	**0.48**
Concerns about the vulnerable	0.23	**0.53**
Concerns about economic damage	0.25	**0.63**
SS loadings	1.30	1.10
Variance (%)	0.26	0.22
Cumulative (%)	0.26	0.48

#### Confirmatory factor analysis

CFA using the maximum likelihood estimation was conducted to confirm the suitability of the two-factor model of concerns about COVID-19 based on the EFA results.

The result of CFA was evaluated using the Tucker-Lewis index (TLI) and comparative fit index (CFI) with cut-offs of ≥ 0.90 and ≥ 0.95 for adequate and good data model fits, respectively. Standardized root mean square residual (SRMR) and root mean square error of approximation (RMSEA) values of ≤ 0.10, ≤ 0.08, and ≤ 0.05 denote acceptable, adequate, and good data-model fits, respectively [32, 36, 37].

The model had a satisfactory fit to the data (χ^2^ = 2913.71 [df = 4], *p* < 0.001, CFI = 0.99, TLI = 0.97, RMSEA = 0.06, SRMR = 0.02). Although the p-value in the chi-squared test was < 0.001, this test has limitations as a measure of model fit because it is sensitive to sample size; larger samples are associated with smaller p-values [38, 39], so we referred to other indices more.

**Fig. 1:**
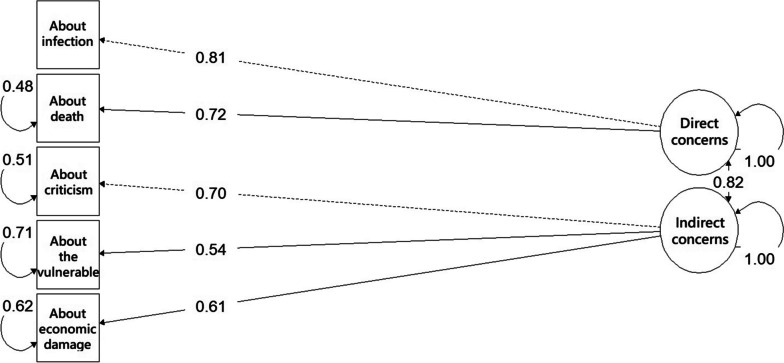
CFA factor loadings

Standardized factor loading values ranged from 0.72 to 0.81 for direct concerns and 0.54 to 0.70 for indirect concerns. Loading values were all significant (*p* < 0.001) (Fig. 1).

#### Item-total correlation test

Table 2 show the results of the item-total correlation test, which was conducted to verify that each item in the direct and indirect concerns categories was representative of its class. If the corrected item-total correlation (C-ITC) is < 0.30, the item makes a small contribution, while if it is > 0.80, it is highly likely to be a duplicate item [40]. To verify the reliability of the items, internal consistency was assessed based on Cronbach’s alpha.

There were no inappropriate items for direct concerns— the C-ITCs of all sub-items were > 0.7. Regarding indirect concerns, the C-ITC values of the items were not as high as those of direct concerns, but were all > 0.5. Also, Cronbach’s alpha values obtained after omitting individual items were similar to that with all items included, so no items needed to be deleted (Table 2). Therefore, it was appropriate to classify concerns about COVID-19 into direct and indirect concerns.

**Table 2:** Sub-categories of direct and indirect concerns

**Direct concerns**	**Mean**	**SD**	**Corrected item-total correlation**	**Cronbach’s ⍺** ** If item is deleted**
About infection	3.94	1.000	0.730	0.943
About death	3.30	1.192	0.771	0.930
Total	3.62	0.977	Cronbach’s alpha = 0.9105	
**Indirect concerns**	**Mean**	**SD**	**Corrected item-total correlation**	**Cronbach’s ⍺** ** If item is deleted**
About criticism	4.01	1.005	0.556	0.820
About the vulnerable	4.08	1.193	0.568	0.830
About economic damage	4.12	1.009	0.582	0.808
Total	4.07	0.822	Cronbach’s alpha = 0.8244	

### Dependent variables and mean concern scores

Table 3, 4, and 5 show the descriptive statistics of sociodemographic, health-related, and COVID-19-related variables, respectively, and the mean values for overall, direct, and indirect concerns. The differences in mean values were all significant (*p* < 0.001).

For sociodemographic variables (Table 3), females had higher mean values for concerns in all categories than males. As age increased, the mean values for concerns in all categories increased. For occupation, the mean values of overall and direct concerns were highest for the unemployed and lowest for white-collar workers. And, the mean values of indirect concerns were higher for pink- and blue-collar workers than for the unemployed. As annual household income increased, the mean values of concerns in all categories decreased. Mean values of concerns in all categories are lower with a higher level of education. Single persons had the lowest mean values of concerns in all categories. By region, mean values of indirect concerns were higher among people residing in Chungcheong and Gangwon provinces, while mean value of direct concerns was lower among residents of Jeju Island.

**Table 3:** Mean concern scores according to sociodemographic variables

Sociodemographic variables	N^a^ (%^b^)	Overall concern scores		Direct concern scores		Indirect concern scores	
		Mean ± SD	*P* value	Mean ± SD	*P* value	Mean ± SD	*P* value
**Sex**							
Male	94,397 (49.52)	3.69 ± 0.78		3.36 ± 0.96		3.90 ± 0.84	
Female	113,709 (50.48)	3.91 ± 0.74	< 0.001	3.67 ± 0.91	< 0.001	4.07 ± 0.80	< 0.001
**Age (y)**							
19–39	47,148 (33.29)	3.67 ± 0.76		3.34 ± 0.92		3.89 ± 0.84	
40–59	73,126 (39.05)	3.78 ± 0.74		3.49 ± 0.91		3.98 ± 0.81	
≥ 60	87,832 (27.66)	3.98 ± 0.78	< 0.001	3.78 ± 0.98	< 0.001	4.11 ± 0.82	< 0.001
**Occupation**							
White collar	39,551 (26.06)	3.69 ± 0.73		3.37 ± 0.88		3.91 ± 0.81	
Pink collar	26,548 (13.69)	3.82 ± 0.75		3.49 ± 0.94		4.03 ± 0.81	
Blue collar	60,688 (22.36)	3.82 ± 0.77		3.51 ± 0.97		4.02 ± 0.82	
Unemployed	81,319 (37.89)	3.86 ± 0.79	< 0.001	3.63 ± 0.97	< 0.001	4.00 ± 0.84	< 0.001
**Annual household Income**							
≤ 1800	59,468 (18.38)	3.92 ± 0.81		3.71 ± 1.01		4.07 ± 0.84	
≤ 3600	57,026 (26.39)	3.83 ± 0.77		3.55 ± 0.96		4.02 ± 0.82	
≤ 6000	53,163 (30.10)	3.78 ± 0.75		3.47 ± 0.92		3.98 ± 0.81	
> 6000	38,449 (25.12)	3.70 ± 0.75	< 0.001	3.40 ± 0.91	< 0.001	3.90 ± 0.82	< 0.001
**Education**							
Middle or low	68,955 (19.31)	4.05 ± 0.77		3.85 ± 0.98		4.18 ± 0.80	
High	60,367 (29.58)	3.83 ± 0.77		3.55 ± 0.95		4.01 ± 0.83	
College or over	78,784 (51.12)	3.69 ± 0.75	< 0.001	3.37 ± 0.90	< 0.001	3.90 ± 0.82	< 0.001
**Marital status**							
Married	130,923 (60.74)	3.86 ± 0.74		3.58 ± 0.93		4.05 ± 0.79	
Separated, divorced, widowed	40,627 (14.43)	3.91 ± 0.80		3.70 ± 0.99		4.05 ± 0.85	
Single	36,556 (24.83)	3.58 ± 0.77	< 0.001	3.26 ± 0.92	< 0.001	3.79 ± 0.86	< 0.001
**Region**							
Metropolitan area	64,646 (48.60)	3.77 ± 0.76		3.48 ± 0.93		3.96 ± 0.82	
Jeolla Province	34,417 (10.19)	3.85 ± 0.79		3.63 ± 0.98		3.99 ± 0.85	
Gyeongsang Province	60,697 (25.81)	3.79 ± 0.77		3.49 ± 0.96		4.00 ± 0.82	
Chungcheong Province	29,075 (10.94)	3.90 ± 0.78		3.63 ± 0.96		4.08 ± 0.82	
Gangwon Province	15,394 (3.26)	3.90 ± 0.78		3.61 ± 1.00		4.10 ± 0.81	
Jeju island	3,877 (1.20)	3.65 ± 0.80	< 0.001	3.28 ± 0.94	< 0.001	3.90 ± 0.88	< 0.001

Range of mean scores: 1–5, Higher scores indicate higher levels of concern

Unit of annual household income: 10,000 KRW

^a^Unweighted

^b^Weighted

Regarding health-related variables (Table 4), respondents with poor subjective health and those with high subjective stress levels had higher mean values for concerns in all categories. Respondents diagnosed with hypertension and diabetes had higher mean values for concerns in all categories than those who did not. Regarding annual unmet healthcare needs, respondents who did not need healthcare services had the lowest mean values for concerns in all categories, and those had unmet healthcare needs had higher mean values for overall and indirect concerns than those who did not.

**Table 4:** Mean concern scores according to health-related variables

Health-related variables	N^a^ (%^b^)	Overall concern scores		Direct concern scores		Indirect concern scores	
		Mean ± SD	*P* value	Mean ± SD	*P* value	Mean ± SD	*P* value
**Subjective health level**							
Good	180,339 (90.62)	3.77 ± 0.76		3.48 ± 0.94		3.97 ± 0.83	
Poor	27,767 (9.38)	4.04 ± 0.78	< 0.001	3.86 ± 1.00	< 0.001	4.15 ± 0.81	< 0.001
**Subjective stress level**							
High	45,814 (25.42)	3.91 ± 0.73		3.60 ± 0.95		4.11 ± 0.78	
Low	162,292 (74.58)	3.76 ± 0.78	< 0.001	3.49 ± 0.95	< 0.001	3.94 ± 0.84	< 0.001
**Hypertension diagnosis**							
Yes	57,998 (20.91)	3.93 ± 0.78		3.71 ± 0.97		4.08 ± 0.82	
No	150,108 (79.09)	3.76 ± 0.76	< 0.001	3.47 ± 0.94	< 0.001	3.96 ± 0.83	< 0.001
**Diabetes diagnosis**							
Yes	24,325 (8.96)	3.96 ± 0.78		3.75 ± 0.99		4.09 ± 0.82	
No	183,781 (91.04)	3.78 ± 0.77	< 0.001	3.49 ± 0.94	< 0.001	3.98 ± 0.83	< 0.001
**Annual unmet healthcare needs**							
Yes	10,497 (4.78)	3.86 ± 0.73		3.54 ± 0.96		4.08 ± 0.79	
No	181,264 (86.02)	3.82 ± 0.77		3.54 ± 0.95		4.00 ± 0.82	
N/A	16,345 (9.20)	3.61 ± 0.79	< 0.001	3.28 ± 0.94	< 0.001	3.82 ± 0.88	< 0.001

Range of mean scores: 1–5, Higher scores indicate higher levels of concern

^a^Unweighted

^b^Weighted

For COVID-19-related variables (Table 5), the greater the changes in daily life, the higher the mean values for concerns in all categories. Moreover, the greater the number of assistants during quarantine, the lower the mean values for concerns in all categories. Respondents who felt that the government’s response to COVID-19 was good had the highest mean values for concerns in all categories. Respondents who believed that the responses of their cities and neighbors to COVID-19 were fair and good had the lowest and highest mean values for concerns in all categories, respectively. Finally, the more appropriate the mass media’s response to COVID-19 was considered to be, the higher the mean values for concerns in all categories.

**Table 5:** Mean concern scores according to COVID-19-related variables

COVID-19 related variables	N^a^ (%^b^)	Overall concern scores		Direct concern scores		Indirect concern scores	
		Mean ± SD	*P* value	Mean ± SD	*P* value	Mean ± SD	*P* value
**Daily life change**							
Severe	58,117 (30.53)	3.94 ± 0.73		3.65 ± 0.94		4.13 ± 0.77	
Moderate	78,571 (38.49)	3.81 ± 0.74		3.53 ± 0.91		4.00 ± 0.80	
Mild or none	71,418 (30.98)	3.64 ± 0.82	< 0.001	3.37 ± 0.99	< 0.001	3.83 ± 0.88	< 0.001
**Numbers of assistants during quarantine**							
None	35,931 (15.60)	3.90 ± 0.79		3.65 ± 0.99		4.07 ± 0.84	
1–2	91,778 (44.87)	3.84 ± 0.75		3.57 ± 0.92		4.02 ± 0.80	
3–5	59,298 (29.39)	3.73 ± 0.76		3.43 ± 0.94		3.94 ± 0.82	
≥ 6	21,099 (10.14)	3.66 ± 0.83	< 0.001	3.33 ± 1.01	< 0.001	3.87 ± 0.89	< 0.001
**Appropriateness of the government’s response**							
Good	152,677 (71.64)	3.83 ± 0.75		3.55 ± 0.94		4.01 ± 0.81	
Fair	41,140 (21.03)	3.72 ± 0.78		3.44 ± 0.95		3.91 ± 0.85	
Poor	14,289 (7.33)	3.73 ± 0.85	< 0.001	3.40 ± 1.07	< 0.001	3.96 ± 0.89	< 0.001
**Appropriateness of the city’s response**							
Good	148,320 (67.69)	3.83 ± 0.76		3.56 ± 0.94		4.01 ± 0.81	
Fair	48,259 (25.78)	3.72 ± 0.77		3.43 ± 0.94		3.92 ± 0.84	
Poor	11,527 (6.53)	3.77 ± 0.84	< 0.001	3.44 ± 1.06	< 0.001	3.99 ± 0.88	< 0.001
**Appropriateness of mass media’s response**							
Good	138,524 (61.55)	3.86 ± 0.76		3.59 ± 0.95		4.04 ± 0.81	
Fair	53,272 (29.04)	3.72 ± 0.76		3.42 ± 0.92		3.91 ± 0.83	
Poor	16,310 (9.41)	3.67 ± 0.81	< 0.001	3.31 ± 1.02	< 0.001	3.90 ± 0.87	< 0.001
**Appropriateness of neighbor’s response**							
Good	152,865 (71.20)	3.83 ± 0.77		3.54 ± 0.95		4.02 ± 0.82	
Fair	47,903 (24.91)	3.72 ± 0.76		3.44 ± 0.93		3.90 ± 0.83	
Poor	7,338 (3.89)	3.80 ± 0.83	< 0.001	3.50 ± 1.03	< 0.001	3.99 ± 0.89	< 0.001

Range of mean scores: 1–5, Higher scores indicate higher levels of concern

^a^Unweighted

^b^Weighted

### Factors influencing direct and indirect concerns about COVID-19

Three multiple regression models were used to analyze each category of concerns. We included sociodemographic variables in model 1; health-related variables in model 2; and COVID-19-related variables in model 3. Before the analysis, multicollinearity between independent variables was analyzed. The variance inflation factor (VIF) values ranged from 1.01 to 1.36 in all models, thus ruling out multicollinearity. The Durbin-Watson values of all models ranged from 1.43 to 1.56, indicating no serious autocorrelation. All results are shown in Table 6 and 7.

**Table 6:** Results of multiple regression analysis of concerns about COVID-19

Independent variables (Ref)	**Overall concerns**			**Direct concerns**			**Indirect concerns**		
	Model 1	Model 2	Model 3	Model 1	Model 2	Model 3	Model 1	Model 2	Model 3
**Sex** (Male)									
Female	0.19 (.00)^***^	0.19 (.00)^***^	0.16 (.00)^***^	0.26 (.00)^***^	0.26 (.00)^***^	0.23 (.00)^***^	0.15 (.00)^***^	0.14 (.00)^***^	0.12 (.00)^***^
**Age (y)** (19–39)									
40–59	-0.04 (.00)^***^	-0.04 (.00)^***^	-0.04 (.00)^***^	0.02 (.01)^**^	0.01 (.01)	0.01 (.01)	-0.07 (.01)^***^	-0.07 (.01)^***^	-0.07 (.01)^***^
≥ 60	0.04 (.01)^***^	0.04 (.01)^***^	0.04 (.01)^***^	0.15 (.01)^***^	0.13 (.01)^***^	0.14 (.01)^***^	-0.04 (.01)^***^	-0.02 (.01)^**^	-0.02 (.01)^**^
**Occupation** (Unemployed)									
White collar	-0.01 (.00)^**^	-0.02 (.00)^***^	0.00 (.00)	-0.06 (.01)^***^	-0.06 (.01)^***^	-0.04 (.01)^***^	0.02 (.01)^**^	0.01 (.01)	0.02 (.01)^***^
Pink collar	0.03 (.01)^***^	0.03 (.01)^***^	0.04 (.01)^***^	-0.05 (.01)^***^	-0.04 (.01)^***^	-0.03 (.01)^***^	0.08 (.01)^***^	0.08 (.01)^***^	0.08 (.01)^***^
Blue collar	0.02 (.00)^***^	0.03 (.00)^***^	0.04 (.00)^***^	-0.03 (.01)^***^	-0.02 (.01)^**^	-0.01 (.01)	0.06 (.01)^***^	0.06 (.01)^***^	0.07 (.01)^***^
**Income** (≤ 1800)									
≤ 3600	0.00 (.01)	0.02 (.01)^**^	0.02 (.01)^***^	-0.01 (.01)	0.01 (.01)	0.01 (.01)^*^	0.01 (.01)	0.02 (.01)^***^	0.02 (.01)^***^
≤ 6000	-0.03 (.01)^***^	-0.01 (.01)	-0.01 (.01)	-0.04 (.01)^***^	-0.01 (.01)	-0.01 (.01)	-0.02 (.01)^***^	-0.01 (.01)	0.00 (.01)
> 6000	-0.08 (.01)^***^	-0.06 (.01)^***^	-0.05 (.01)^***^	-0.07 (.01)^***^	-0.04 (.01)^***^	-0.03 (.01)^***^	-0.09 (.01)^***^	-0.07 (.01)^***^	-0.06 (.01)^***^
**Education** (College or over)									
High	0.07 (.00)^***^	0.06 (.00)^***^	0.06 (.00)^***^	0.09 (.01)^***^	0.08 (.01)^***^	0.08 (.01)^***^	0.05 (.00)^***^	0.05 (.00)^***^	0.05 (.00)^***^
Middle or low	0.19 (.01)^***^	0.17 (.01)^***^	0.17 (.01)^***^	0.21 (.01)^***^	0.18 (.01)^***^	0.18 (.01)^***^	0.17 (.01)^***^	0.16 (.01)^***^	0.16 (.01)^***^
**Marital status** (Single)									
Separated, divorced, and widowed	0.14 (.01)^***^	0.12 (.01)^***^	0.11 (.01)^***^	0.13 (.01)^***^	0.11 (.01)^***^	0.10 (.01)^***^	0.15 (.01)^***^	0.13 (.01)^***^	0.11 (.01)^***^
Married	0.23 (.01)^***^	0.22 (.00)^***^	0.20 (.00)^***^	0.20 (.01)^***^	0.18 (.01)^***^	0.17 (.01)^***^	0.25 (.01)^***^	0.24 (.01)^***^	0.22 (.01)^***^
**Region** (Metropolitan area)									
Jeolla Province	0.03 (.01)^***^	0.04 (.01)^***^	0.04 (.01)^***^	0.10 (.01)^***^	0.10 (.01)^***^	0.09 (.01)^***^	-0.01 (.01)	0.00 (.01)	0.00 (.01)
Gyeongsang Province	-0.01 (.00)^*^	0.00 (.00)	0.02 (.00)^***^	-0.04 (.00)^***^	-0.03 (.00)^***^	-0.01 (.00)	0.01 (.00)^*^	0.02 (.00)^***^	0.04 (.00)^***^
Chungcheong Province	0.10 (.01)^***^	0.10 (.01)^***^	0.11 (.01)^***^	0.11 (.01)^***^	0.12 (.01)^***^	0.13 (.01)^***^	0.09 (.01)^***^	0.10 (.01)^***^	0.11 (.01)^***^
Gangwon Province	0.08 (.01)^***^	0.09 (.01)^***^	0.09 (.01)^***^	0.07 (.01)^***^	0.07 (.01)^***^	0.08 (.01)^***^	0.09 (.01)^***^	0.10 (.01)^***^	0.11 (.01)^***^
Jeju Island	-0.14 (.02)^***^	-0.14 (.01)^***^	-0.15 (.01)^***^	-0.22 (.02)^***^	-0.22 (.02)^***^	-0.24 (.02)^***^	-0.08 (.02)^***^	-0.08 (.02)^***^	-0.09 (.02)^***^
**Subjective health** (Good)									
Poor		0.09 (.01)^***^	0.09 (.01)^***^		0.16 (.01)^***^	0.15 (.01)^***^		0.05 (.01)^***^	0.05 (.01)^***^
**Subjective stress** (Low)									
High		0.16 (.00)^***^	0.15 (.00)^***^		0.14 (.00)^***^	0.13 (.00)^***^		0.18 (.00)^***^	0.16 (.00)^***^

Standard errors are presented in parentheses. Unit of annual household income: 10,000 KRW

Model 1: Sociodemographic variables

Model 2: Sociodemographic and health-related variables

Model 3: Sociodemographic, health-related, and COVID-19 related variables

^*^p < 0.5

^**^p < 0.01

^***^p < 0.001

**Table 7:** Results of multiple regression analysis of concerns about COVID-19 (continued)

Independent variables (Ref)	**Overall concerns**			**Direct concerns**			**Indirect concerns**		
	Model 1	Model 2	Model 3	Model 1	Model 2	Model 3	Model 1	Model 2	Model 3
**Hypertension** (No)									
Yes		0.02 (.00)^***^	0.03 (.00)^***^		0.04 (.01)^***^	0.04 (.01)^***^		0.01 (.00)^*^	0.01 (.00)^**^
**Diabetes** (No)									
Yes		0.03 (.01)^***^	0.04 (.01)^***^		0.06 (.01)^***^	0.07 (.01)^***^		0.01 (.01)^*^	0.02 (.01)^**^
**Unmet healthcare needs** (n/a)									
No		0.12 (.01)^***^	0.10 (.01)^***^		0.13 (.01)^***^	0.12 (.01)^***^		0.10 (.01)^***^	0.09 (.01)^***^
Yes		0.11 (.01)^***^	0.10 (.01)^***^		0.09 (.01)^***^	0.07 (.01)^***^		0.13 (.01)^***^	0.11 (.01)^***^
**Daily life changes** (Mild or none)									
Moderate			0.16 (.00)^***^			0.16 (.00)^***^			0.17 (.00)^***^
Severe			0.28 (.00)^***^			0.27 (.01)^***^			0.29 (.00)^***^
**Numbers of assistants during quarantine** (≥ 6)									
3–5			0.06 (.01)^***^			0.06 (.01)^***^			0.05 (.01)^***^
1–2			0.13 (.01)^***^			0.15 (.01)^***^			0.11 (.01)^***^
None			0.13 (.01)^***^			0.15 (.01)^***^			0.11 (.01)^***^
**Appropriateness of the government’s response** (Good)									
Fair			-0.03 (.01)^***^			-0.02 (.01)^***^			-0.03 (.01)^***^
Poor			-0.04 (.01)^***^			-0.05 (.01)^***^			-0.03 (.01)^***^
**Appropriateness of the city’s response** (Good)									
Fair			-0.04 (.01)^***^			-0.04 (.01)^***^			-0.03 (.01)^***^
Poor			0.01 (.01)			-0.01 (.01)			0.02 (.01)^*^
**Appropriateness of media’s response** (Good)									
Fair			-0.08 (.00)^***^			-0.10 (.01)^***^			-0.07 (.00)^***^
Poor			-0.16 (.01)^***^			-0.22 (.01)^***^			-0.12 (.01)^***^
**Appropriateness of neighbor’s response** (Good)									
Fair			-0.04 (.00)^***^			-0.01 (.01)			-0.06 (.00)^***^
Poor			0.03 (.01)^***^			0.07 (.01)^***^			0.01 (.01)

Standard errors are presented in parentheses

Model 1: Sociodemographic variables

Model 2: Sociodemographic and health-related variables

Model 3: Sociodemographic, health-related, and COVID-19 related variables

^*^p < 0.5

^**^p < 0.01

^***^p < 0.001

In model 1, we included sex, age, occupation, annual household income, education, marital status, and region. Females had higher concerns in all categories than males, especially direct concerns (B = 0.26). Regarding age, compared with the early adulthood group (19–39 years), overall and indirect concerns were lower in the middle adulthood group (40–59 years) (B = -0.04, and -0.07, respectively). In the elderly group (≥ 60 years), overall and direct concerns increased more than in the early adulthood group (B = 0.04, and 0.15, respectively), whereas indirect concerns decreased (B = -0.04). Regarding occupation, direct concerns tended to decrease in all workers compared to the unemployed, whereas indirect concerns increased in all workers, especially in pink- and blue-collar ones (B = 0.08, and 0.06, respectively). Regarding annual household income, compared with the lowest income group (≤ 1,800, unit = 10,000 KRW), concerns in all categories decreased as income increased, except for the second income quantile (≤ 3600). A lower level of education was associated with greater concerns in all categories. Regarding marital status, concerns in all categories decreased in single people, and married people showed a greater increase in concerns than those who were separated, divorced, or widowed. Compared to metropolitan areas, direct concerns increased among those residing in Chungcheong province (B = 0.11), and indirect concerns increased among those residing both Chungcheong and Gangwon provinces (B = 0.09).

Model 2 further included subjective health level, subjective stress level, hypertension, diabetes, and annual unmet healthcare needs. Direct concerns increased especially in respondents with poor subjective health (B = 0.16), and indirect concerns increased especially in those with high subjective stress levels (B = 0.18). Respondents with hypertension and diabetes had greater concerns in all categories than those without those conditions, especially direct concerns (B = 0.04, and 0.06, respectively). Compared to respondents who did not need healthcare services, those who did had higher concerns in all categories. Among them, respondents with unmet healthcare needs had greater indirect concerns than those without (B = 0.13), and vice versa for direct concerns (B = 0.13).

Model 3 additionally included daily life changes related to COVID-19, the number of assistants during COVID-19 quarantine, and the appropriateness of the COVID-19 response of the government, city, mass media, and neighbors. The more severe the changes in daily life related to COVID-19, the greater the concerns in all categories. Compared to respondents with ≥ 6 assistants during COVID-19 quarantine, those with fewer assistants had greater concerns, although there was no difference in concerns between those with 1–2 versus 0 assistants. Furthermore, the less appropriate the perceived response of the government and mass media to COVID-19, the lower the level of concerns. Indirect concerns of respondents who believed that their city’s response to COVID-19 was poor were greater compared to those who believed it was good (B = 0.02). Direct concerns increased among respondents who believed that their neighbors’ response to COVID-19 was poor compared to those who believed it was good (B = 0.07).

### Relative importance of factors influencing concerns about COVID-19

**Fig. 2:**
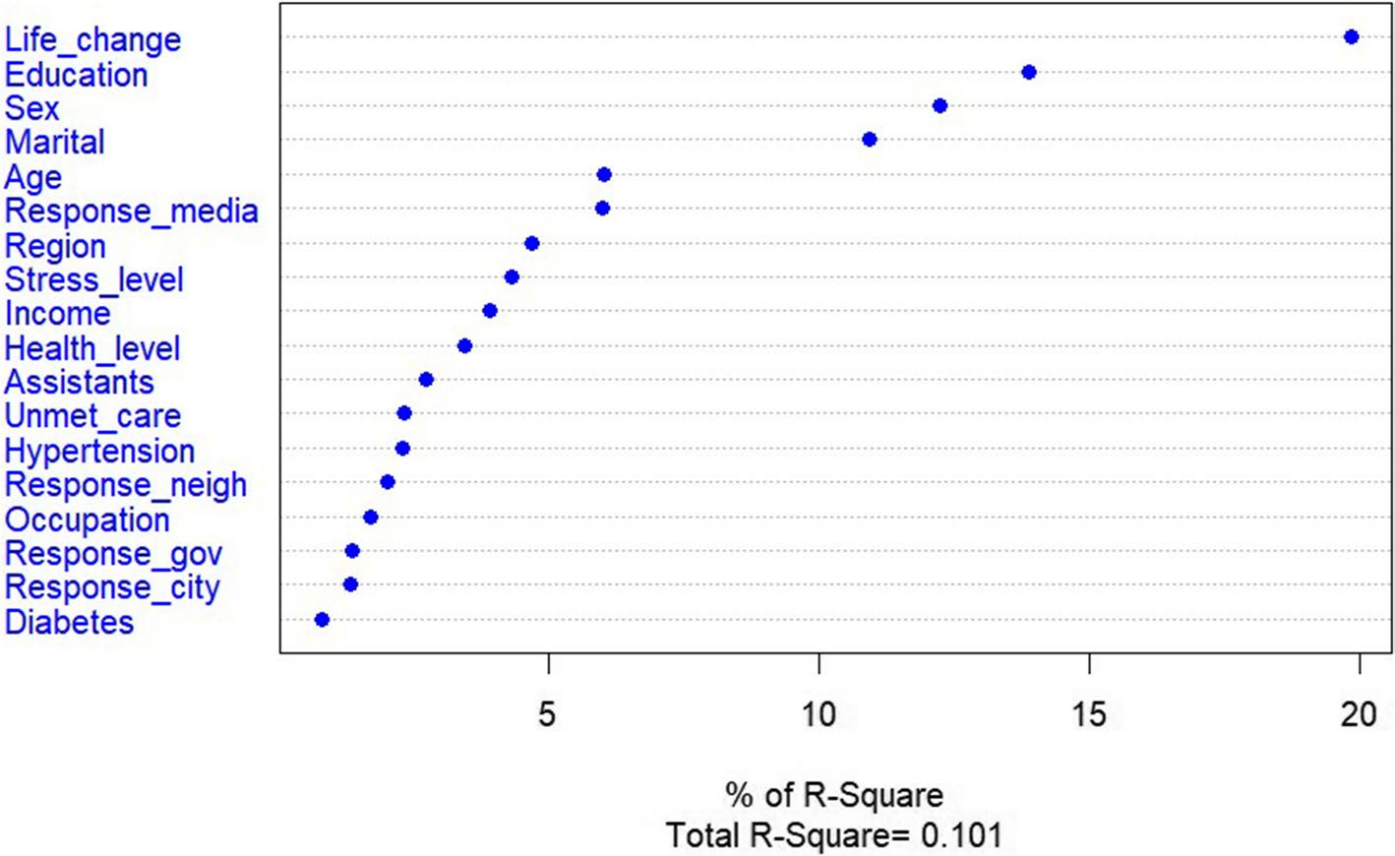
Predictors of overall concerns

Among the factors influencing overall concerns, daily life change was the most important, followed by education level, sex, and marital status (Fig. 2).

**Fig. 3:**
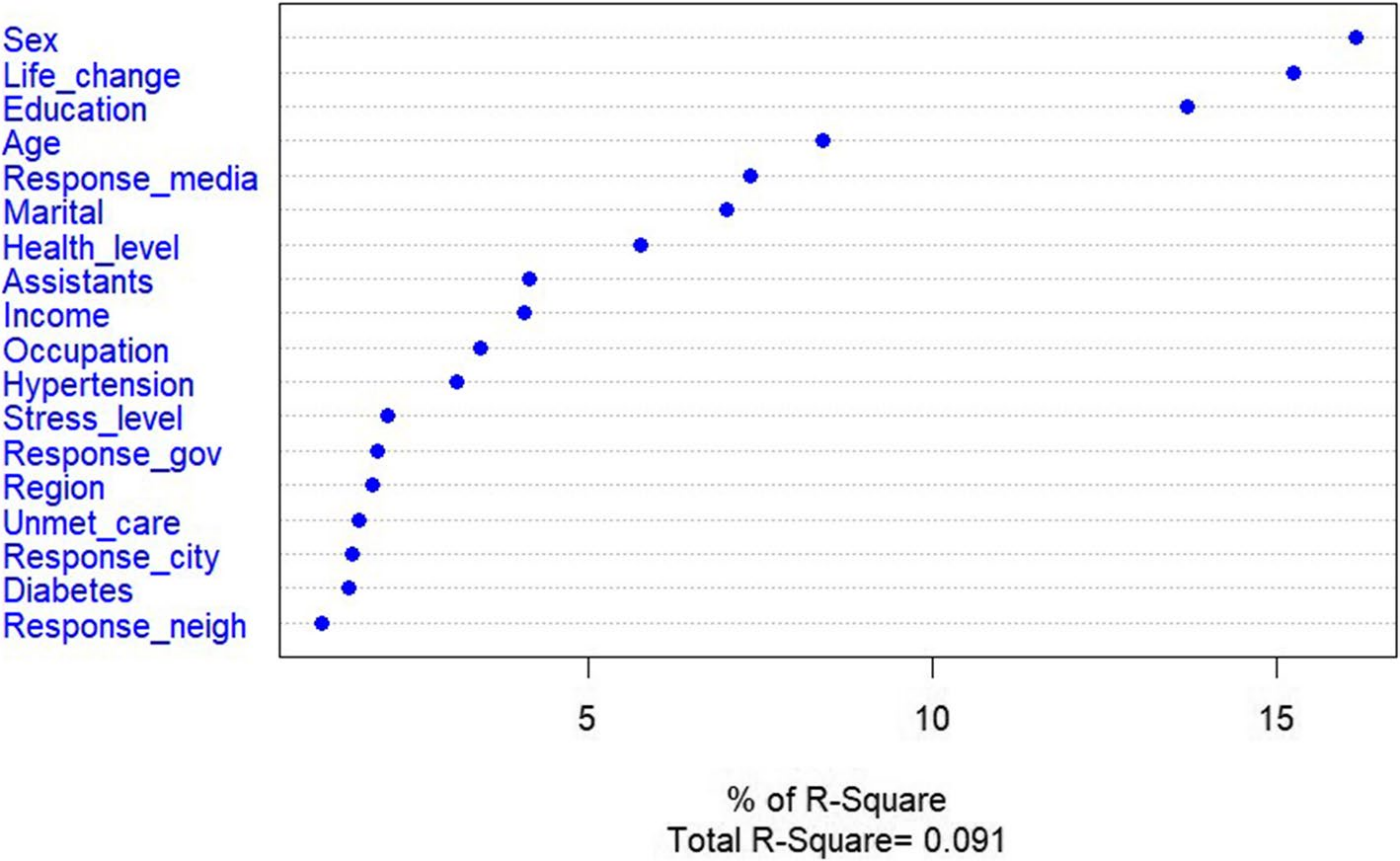
Predictors of direct concerns

Among the factors influencing direct concerns, sex, daily life change, and education level were the most important. Importance decreased in the order of age, appropriateness of the COVID-19 response of mass media, marital status, and subjective health level (Fig. 3).

**Fig. 4:**
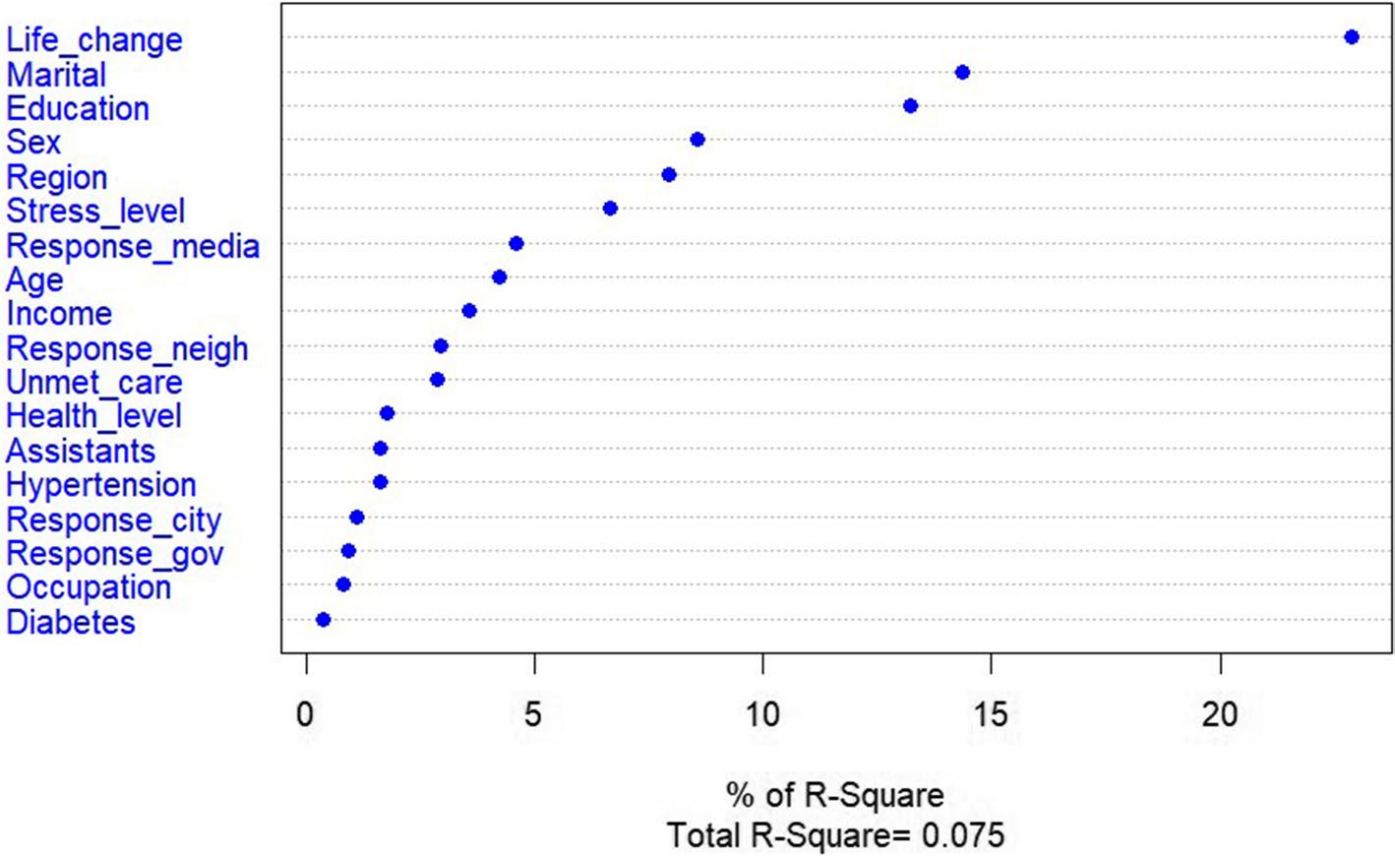
Predictors of indirect concerns

Among the factors influencing indirect concerns, daily life change, marital status, and educational level were the most important. Importance decreased in the order of sex, region, and subjective stress level (Fig. 4).

## Discussion

Concern is an important indicator of the mental health effects of COVID-19; it serves acts as a predictor of mental disorders and acted as a proxy of mental health in the early stages of COVID-19 [23–25]. Many studies have evaluated factors related to psychological concerns, but few have compared the impacts of the direct and indirect damage caused by COVID-19 on such concerns. Accordingly, we aimed to compare the impacts of direct and indirect damage from the pandemic on psychological concerns and identify relatively vulnerable groups. Psychological concerns about COVID-19 were divided into direct and indirect categories, and the distribution of concerns differs across populations. The key findings of this study are described in more detail below.

Some of our findings are consistent with previous reports. Direct and indirect concerns were greater in respondents with low incomes and education levels, and in those who reported major changes in their daily life. Concerns increased as income and education level decreased, because disadvantaged socioeconomic status is a risk factor for mental health problems such as anxiety and worry [2, 41, 42]. We evaluated the magnitude of changes in daily life relative to the pre-pandemic period, so our findings are consistent with reports that the changes brought by lockdown and social distancing negatively affected mental health [4, 43–45].

Direct concerns were especially prevalent among females, respondents with poor subjective health, those with hypertension or diabetes, those with few assistants during quarantine, and those who believed that their neighbor’s response to COVID-19 was inappropriate. Direct concerns were more prevalent among females, consistent with a report that females have more negative expectations and greater levels of fear about health-related outcomes of COVID-19 than males [46]. Concerns were greater among respondents with hypertension or diabetes, especially direct concerns. Indeed, patients with chronic diseases in a previous study also had more concerns about COVID-19 [47]. Direct concerns were greater among our respondents who had fewer than two assistants during the COVID-19 quarantine. In college students, psychological concerns were less serious when they could ask someone for help during the COVID-19 pandemic [48]. Direct concerns were greater among our respondents who believed that their neighbor’s response to COVID-19 was inappropriate, likely because contact with neighbors or co-workers who are not appropriately responding to COVID-19 can increase the risk of infection.

Indirect concerns were greater among our married respondents, those who felt their city’s response to COVID-19 was inappropriate and residents of Chungcheong and Gangwon provinces. Concerns were more prevalent among married respondents, particularly indirect concerns, which may be because they tended to have young children vulnerable to disease. The previous study also suggested that the COVID-19 worry scores were elevated in married and cohabiting individuals [49]. Because the city is responsible for policies related to indirect damage from the pandemic, including the COVID-19 Emergency Relief Fund and support schemes for small business owners, an inappropriate response therefrom would likely have heightened indirect concerns. In mid-August 2020, there was a significant increase in confirmed COVID-19 cases in Gangwon and Chungcheong provinces of Korea, primarily attributed to the use of sports facilities, religious venues, and urban rallies, which led to an escalation in the social distancing policy [50]. Given that this period coincides with the data collection period of this study, it is likely to have influenced the concerns of residents.

There were some intriguing findings in this study in relation to occupation and unmet healthcare needs. Direct concerns were greater among the unemployed respondents, while indirect concerns were greater among those with jobs. This can be attributed to negative effects of the pandemic on workers’ economic activity. In addition, the indirect concerns of pink- and blue-collar workers were particularly high, probably because they are more likely to be employed as non-regular workers than white-collar workers, and because non-regular workers were more likely to experience involuntary unemployment and a decline in income than regular workers during the pandemic [51]. Among our respondents who needed healthcare services over the past year, direct concerns were greater in those who did not experience unmet healthcare needs, while indirect concerns were greater in respondents who experienced unmet healthcare needs. In another Korean study, unmet healthcare needs were lower in individuals who feared that COVID-19 could be fatal [52], indicating that those with few direct concerns may be more likely to have unmet healthcare needs.

Unexpectedly, direct and indirect concerns were greater among our respondents who believed that the responses of the government and mass media to COVID-19 were appropriate. In the early stages of the pandemic, the Korean government implemented mandatory quarantine and social distancing, and prohibited large-scale gatherings. In particular, the Korean government enacted highly proactive measures in response to the occurrence of mass infections, including nationwide school closures, the transition of religious gatherings to online platforms, and the prohibition of operating all clubs and bars within Seoul, in order to prevent the spread of infections [53]. This quarantine system was highly valued both domestically and internationally [54]. In March 2020, South Korea's quarantine strategy garnered attention, and it was during this time that the term 'K-Quarantine' was first explicitly mentioned in media articles. This marked the beginning of national branding for the country's quarantine efforts, and the term 'K-Quarantine' was also used for government promotion, news articles, web pages, and more. It was also evaluated as a source of national pride [54]. However, because most of the government’s responses were aimed at reducing social contact, psychological concerns would likely have increased even among people who believed that the policies were appropriate. Under the assumption that the respondents who believed that the mass media responded appropriately to COVID-19 have more access to the media, our finding is consistent with a report of a bi-directional association between consumption of media related to COVID-19 and worry [55]. Indeed, numerous instances of misinformation and rumors related to COVID-19 circulated through mass media [22], leading to stigmatization and prejudice against specific groups [56, 57]. It was also suggested that these events fueled people's anger and distrust [58]. Media consumption could be a maladaptive coping strategy that increases worry.

This study presented new results that were not previously addressed. First, Psychological concerns about COVID-19 can be classified as direct and indirect concerns. We performed EFA, CFA, and C-ITC to confirm the validity of this classification. Although EFA has been controversial because of its complexity, few other statistical methods are suitable for such classification [34].

Second, significant differences in direct and indirect concerns were found between age groups, providing valuable insights. Overall concern was higher among the elderly than those in early adulthood, consistent with an analysis of data from Daegu City collected in 2020 [59]. Moreover, direct concerns increased significantly among our elderly respondents compared to those in early adulthood, whereas indirect concerns were greater in the latter group. This may be because younger people tend to be more engaged in social and economic activities, and would thus be concerned about the impacts of COVID-19 and quarantine on such activities. Previous studies also suggested that younger age is associated with increased economic fear [60], while the elderly are more concerned about COVID-19-related infections and deaths [7]. Therefore, younger and older persons are vulnerable to different types of damage.

Third, by assessing the relative importance of variables associated with direct and indirect concerns, we identified problems that should be prioritized by policymakers. Daily life changes related to COVID-19 were strongly related to overall, direct, and indirect concerns; strict policies such as social distancing and lockdown have a major psychological impact. Daily life changes were particularly strongly associated with indirect concerns, likely because of their association with decreases in income [61] and social contact [62], for example. The perceived appropriateness of the mass media response to COVID-19 was an important variable in our study; the mass media exerts a major influence during public health crises. Previous researches reported that when the news is biased and misleading, poor physical and mental health outcomes can result [63–65].

### Limitations

Our study had several limitations. First, a trend of change over time or causal relationships could not be confirmed because of the cross-sectional design. Therefore, follow-up studies using longitudinal data are needed. Second, we evaluated psychological concerns in the early stages of the COVID-19 pandemic using 2020 Community Health Survey data, which precluded evaluation of the long-term effects of the COVID-19 pandemic. Third, psychological concerns may have exerted positive effects, such as encouraging activities preventing the rapid spread of COVID-19. However, we treated the concerns only as potential risk factors for poor mental health, thereby limiting the interpretability of the findings. Fourth, some antecedents of indirect concerns may have been disregarded, which could reduce the generalizability of our findings.

Despite these limitations, our study is meaningful because it assessed the associations of the direct and indirect damage caused by the COVID-19 pandemic with psychological concerns in the early stages of COVID-19. We evaluated the vulnerability of various groups to different types of damage, which is important because it provides a basis for the need for individualized interventions for future pandemics and public health crises. Previous studies suggested that major COVID-19-related worries include serious illness, infecting others, death, medical services, economic recession, unemployed, and reduced social contact [66], and major COVID-19-related concerns include reduced social contact, childcare, family, everyday life, paid work, and the economy [26]. Because the causes of psychological concerns presented in this study are similar to those of previous studies, there seems to be no serious problem in generalizing our results.

### Practical implications

Despite noted limitations, our findings have several practical implications. First, individual interventions are needed because different groups are vulnerable to different types of damage. For example, persons living alone and those with small social networks are more vulnerable to direct damage, so supportive policies during quarantine are more required. Policymakers should focus on the elderly and people in early adulthood with regard to direct and indirect damage, respectively, and the importance of mental health interventions for younger people should not be underestimated.

Second, the relative importance of variables influencing concerns should be considered. For example, sex and marital status had a major influence on direct and indirect concerns, respectively. Married women, who may be raising young children, appear to be particularly vulnerable to psychological damage from COVID-19. In other studies, it has been also reported that the health-related quality of life of pregnant women was compromised during the pandemic [67]. So, policymakers should pay more attention to them and consider measures such as providing psychological support services tailored to their needs. Because policies that interfere with daily life influence both direct and indirect concerns, they should be applied with caution, and their necessity should be continuously reevaluated. In addition, the mass media’s response to COVID-19 has a major impact on direct and indirect concerns; the media must provide accurate information during pandemic, and proper regulation of unreliable and inappropriate news is needed.

## Conclusion

Evaluating direct and indirect concerns is important and meaningful. In this study, concerns caused by direct and indirect damages of the pandemic differed according to population characteristics. The relative importance of factors influencing direct and indirect concerns was similar for daily life changes and appropriateness of the COVID-19 response of mass media, and differed for sex and marital status. Our findings can be used to prioritize psychological interventions and policies for future pandemics. Tailored interventions to improve mental health and prevent negative psychological concerns are needed.

## Data Availability

The datasets used and/or analyzed during the current study are available from the corresponding author on reasonable request.
